# Smoldering type adult T‐cell leukemia/lymphoma effectively treated with mogamulizumab (anti‐CC chemokine receptor 4 monoclonal antibody)—A case report

**DOI:** 10.1002/ccr3.2155

**Published:** 2019-04-16

**Authors:** Yotaro Nishikawa, Kosuke Mochida, Tamaki Kubo, Nagako Horikawa, Rieko Nemoto, Masahiro Amano

**Affiliations:** ^1^ Department of Dermatology, Faculty of Medicine University of Miyazaki Miyazaki Japan

**Keywords:** adult T-cell leukemia/lymphoma, CC chemokine receptor 4, graft-vs-host disease, mogamulizumab, smoldering type

## Abstract

The use of mogamulizumab needs careful consideration because of severe adverse reactions such as graft‐vs‐host disease. However, refractory specific skin lesions of smoldering type adult T‐cell leukemia/lymphoma can be effectively treated with mogamulizumab when patients have no opportunity to receive hematopoietic stem cell transplantation like our case.

## 1 INTRODUCTION

Adult T‐cell leukemia/lymphoma (ATLL) is an endemic disease in southwest Japan and the Caribbean basin caused by human T‐lymphotropic virus type 1 (HTLV‐1).^1^ Skin lesions are treated with skin directed therapy containing topical steroid, ultraviolet, surgery, and radiation.^2^ Oral etretinate, or low‐dose etoposide and prednisolone are sometimes selected.^3^, ^4^ Anti‐CC chemokine receptor 4 (CCR4) monoclonal antibody mogamulizumab is also available. We report a case of effectively treated skin lesions of smoldering type ATLL with mogamulizumab.

## 2 CASE REPORT

**Figure 1:**
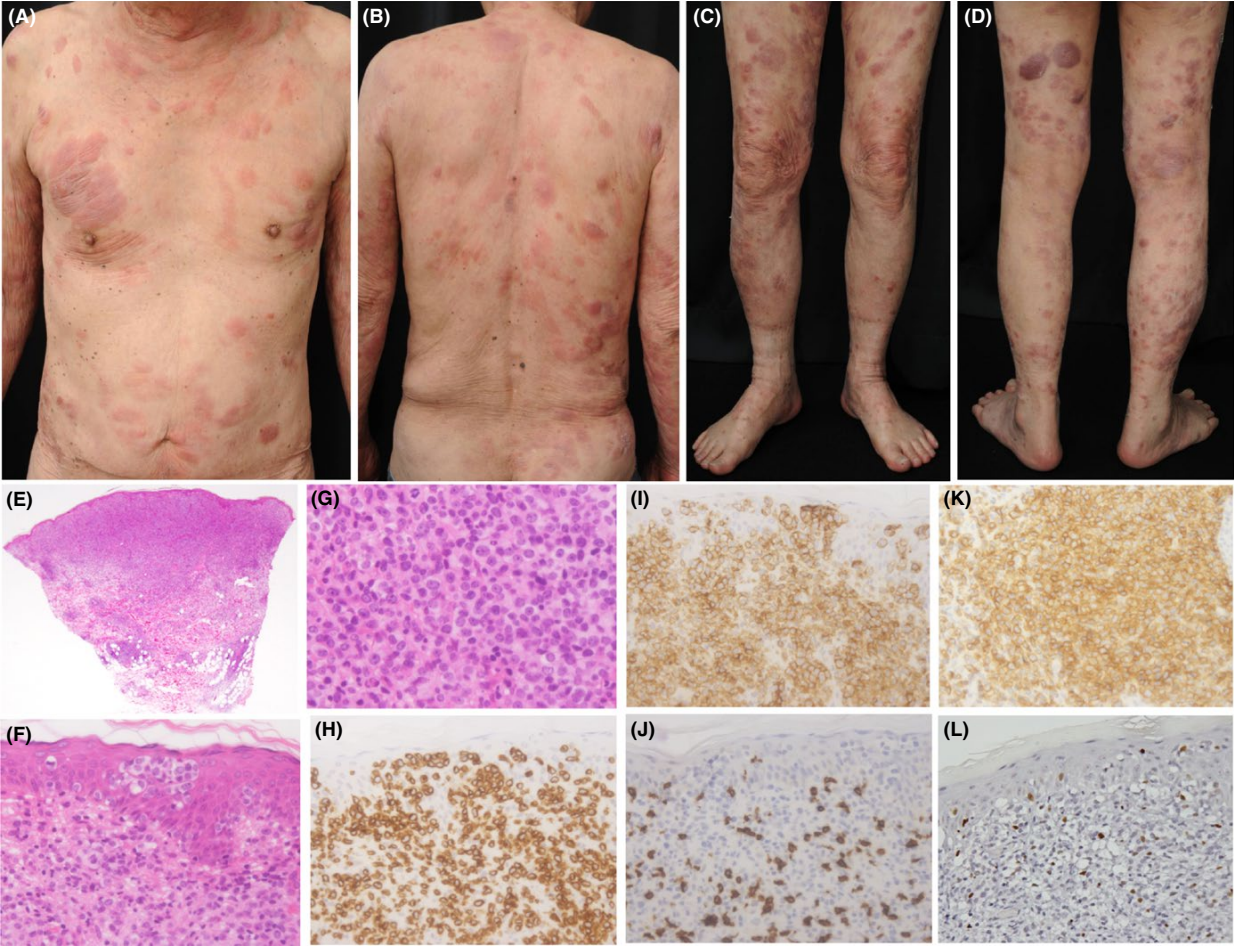
Clinical and histopathological features before administration of mogamulizumab. A‐D, Erythematous plaques and tumors were diffusely observed on the patient's trunk and extremities. E, A dense infiltration of atypical lymphoid cells in the dermis (hematoxylin‐eosin, ×12.5). F and G, Small‐to‐medium‐sized pleomorphic cells with epidermotropism and Pautrier's microabscess (hematoxylin‐eosin, ×400). Atypical cells were (H) CD3^+^ (×400), (I) CD4^+^ (×400), (J) CD8^−^ (×400), and (K) CCR4^+^ (×400). L, Foxp3^+^ cells were observed among atypical cells

A 77‐year‐old man with smoldering type ATLL had been treated for specific skin lesions. He had been also treated for diabetes mellitus with oral hypoglycemic agents. Erythema progressed to plaques and tumors in spite of treatment with topical corticosteroids, narrow‐band ultraviolet B, and oral etretinate. He received electron radiation therapy followed by oral prednisolone and low‐dose etoposide. We stopped prednisolone and etoposide because of severe stomatitis. Although there were increasing multiple plaques and tumors on his trunk and extremities (Figure 1A‐D), progression from smoldering to acute subtype did not occur. Histopathologically, a dense infiltration of small‐to‐medium‐sized pleomorphic lymphoid cells was observed in the dermis with prominent epidermotropism (Figure 1E‐G). Infiltrating cells were CD3^+^, CD4^+^, CD8^−^, CD79a^−^, and CCR4^+^ (Figure 1H‐K). Foxp3^+^ cells were observed among atypical cells (Figure 1L). Although he was elderly, he had no problem with hematological parameters and liver function test: hemoglobin 13.3 g/L, White blood cell count (WBC) 4.6 × 10^9^/L, neutrophils 3.8 × 10^9^/L, lymphocytes 0.33 × 10^9^/L, monocytes 0.38 × 10^9^/L, eosinophils 0.04 × 10^9^/L, basophils 0.02 × 10^9^/L, platelet 221 × 10^9^/L, aspartate aminotransferase (AST) 21 IU/L, and alanine aminotransferase (ALT) 21 IU/L. Blood examination showed mild renal dysfunction: blood urea nitrogen (BUN) 22.7 mg/dL, creatinine 1.06 mg/dL, and estimated glomerular filtration rate (eGFR) 52.3 mL/min/1.73 m^2^. We intended to inject mogamulizumab 1.0 mg/kg, once weekly for 8 weeks.

**Figure 2:**
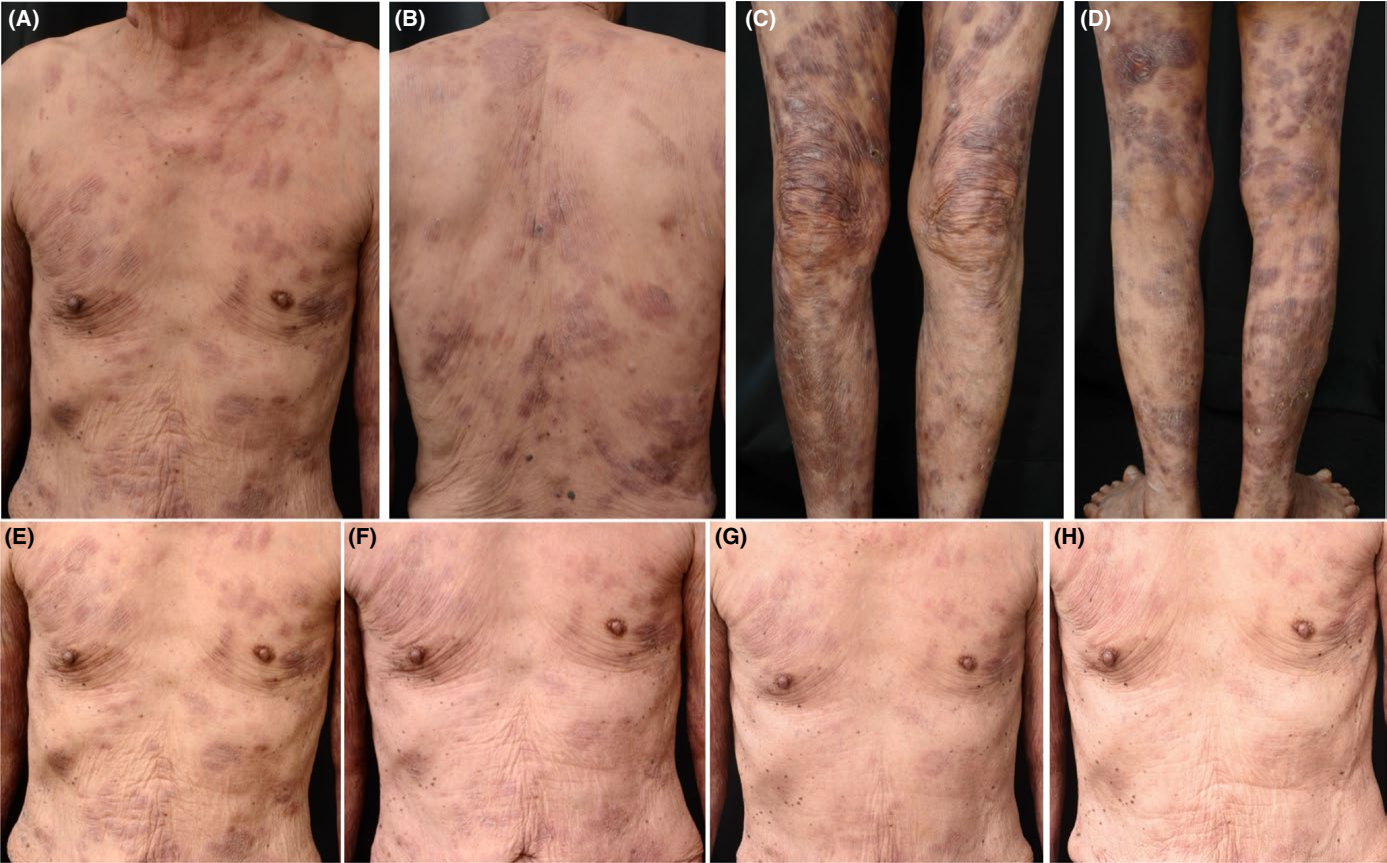
A‐D, 2 d after the first administration of mogamulizumab. Plaques and tumors on the patient's trunk and extremities became flattening and dark reddish‐brown. A week after (E) the first, (F) the third, (G) the fifth, (H) the seventh administration of mogamulizumab

Two days later from the first mogamulizumab administration, plaques and tumors became flattening and dark reddish‐brown (Figure 2A‐D). Skin lesions continued to be improved during treatment period (Figure 2E‐H). Modified Severity‐Weighted Assessment Tool (mSWAT) score was improved 70 (before the first infusion) to 34 (after the second infusion). As more than 50% of skin lesions were improved, we considered partial response (PR) was achieved. Blood examination revealed normal hematological parameters and liver function during and after the mogamulizumab treatment: hemoglobin 13.5 g/L, WBC 6.5 × 10^9^/L, platelet 300 × 10^9^/L, AST 16 IU/L, ALT 15 IU/L after the first infusion, and hemoglobin 12.3 g/L, WBC 5.7 × 10^9^/L, platelet 237 × 10^9^/L, AST 19 IU/L, ALT 13 IU/L after the final infusion. Renal function was not exacerbated: BUN 15.1 mg/dL, creatinine 1.11 mg/dL, eGFR 49.8 mL/min/1.73 m^2^ after the first infusion, and BUN 22.2 mg/dL, creatinine 1.01 mg/dL, eGFR 55.2 mL/min/1.73 m^2^ after the final infusion.

**Figure 3:**
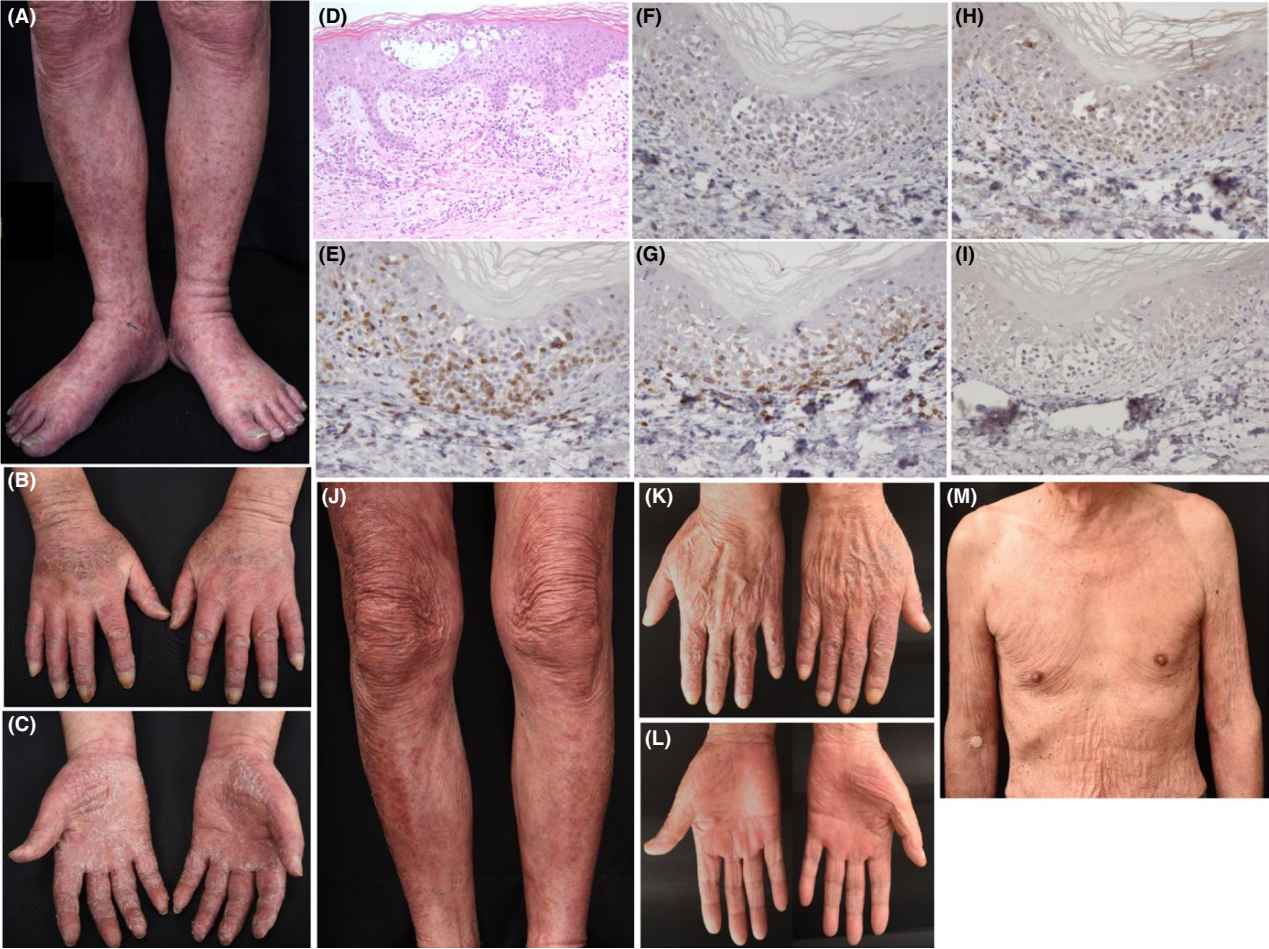
A‐K, Clinical and histopathological features 19 wk after the first administration of mogamulizumab. A, Multiple erythema, papules, and purpuras on the patient's lower legs and foots. B and C, Erythema and cracks on his hands. D, Spongiosis and spongiotic bullas in the epidermis. Lymphocytes, eosinophils, and erythrocytes in the upper dermis (hematoxylin‐eosin, ×200). Lymphocytes were (E) CD3^+^ (×400), (F) CD4^−^ (×400), (G) CD8^+^ (×400), (H) granzyme B^+^ (partially) (×400), and (I) Foxp3^+^ (slightly) (×400). J‐M, 5 wk after starting oral prednisolone for spongiotic dermatitis (24 wk after the first mogamulizumab administration). J, Papules and purpuras on his legs diminished, but erythema was still observed. K and L, Skin lesions on his hands disappeared. M, No skin lesions on his trunk

Erythema and cracks on his hands, and multiple erythema, papules, and purpuras on his lower legs appeared 19 weeks later from the first mogamulizumab treatment (Figure 3A‐C). Histopathologically, spongiosis in the epidermis, liquefactive degeneration of basal cells, and lymphocytes, eosinophils, and erythrocytes in the upper dermis was observed (Figure 3D). Lymphocytes were CD3^+^, CD4^−^, CD8^+^, CD79a^−^, granzyme B^+^ (partially), perforin^−^, TIA‐1^−^, and Foxp3^+^ (slightly) (Figure 3E‐I). We considered his skin lesions as spongiotic dermatitis, not specific skin lesions of ATLL. Spongiotic dermatitis was improved by oral prednisolone 10 mg/d. Only erythema on his legs remained (Figure 3J‐M). The mSWAT score was 24, and PR was maintained.

## 3 DISCUSSION

Mogamulizumab highly enhances antibody‐dependent cellular cytotoxicity (ADCC) of natural killer cells by binding to CCR4 expressed on tumor cells.^5^ Although Mogamulizumab can be used for any subtypes of CCR4 positive ATLL, most cases are for aggressive type.^6^ Efficacy of mogamulizumab for skin lesions is comparatively high. Responses according to disease sites are 74.5%‐100% for blood, 57.7%‐63% for skin, and 25%‐31.5% for nodal and extranodal lesions.^7^, ^8^

Acute graft‐vs‐host disease (GVHD) after allogeneic hematopoietic stem cell transplantation (allo‐HSCT) and cutaneous adverse reactions (CARs) is major adverse events.

Mogamulizumab depletes not only tumor cells but also Foxp3^+^ regulatory T cells (Tregs) and T cells with Th2 phenotype (Th2‐cells) because they also express CCR4 on their surfaces.^9^ Depletion of Tregs enhances antitumor immunity, on the other hand, it may cause severe complications such as GVHD.^9^ Reduction of Th2‐cells results in shifting the Th1/Th2 balance to the Th1 axis, and it might enhance tissue damage through GVHD.^9^ It spends 4 months until the number of Tregs return to baseline level after the last mogamulizumab administration.^10^ The rates of acute GVHD are 65.7% for patients who received allo‐HSCT within 90 days after the last administration of mogamulizumab, and 28.6% after over 91 days.^9^ The shorter interval (<3 months) between the last administration of mogamulizumab and allo‐HSCT is highly associated with severe GVHD.^10^ In our case, the patient was 77 years old (>70 years old) and had no chance to receive allo‐HSCT due to his age even if his illness would have unfortunately become crisis. Therefore, we selected mogamulizumab treatment with not being afraid of the possibility of GVHD.

The frequency of all CARs is 34.3%, and the serious one is 10.7%.^6^ Rash, erythema, and pruritus are common CARs.^6^ Stevens‐Johnson syndrome (SJS) and toxic epidermal necrosis (TEN) are reported in 0.8% and 0.6%, respectively.^6^ Histological features of CARs are spongiosis in the epidermis, liquefactive degeneration of basal cells, and heavy lymphocyte infiltration in the upper dermis.^11^ Infiltrating lymphocytes are positive for CD3, CD8, granzyme B, perforin, and TIA‐1.^11^ The overall response rate (ORR) and the overall survival (OS) are better in patients with CARs.^8^, ^11^ The ORRs for the patients with and without CARs are 78%‐86% and 37%‐47%, respectively.^6^, ^11^ The OS is significantly longer in patients who experienced CARs (15.7 months) than ones who did not (5.4 months).^8^ In our case, PR was achieved, and spongiotic dermatitis with activated cytotoxic T‐cells positive for CD8 and granzyme B appeared 10 weeks after from the last administration of mogamulizumab. Furthermore, Foxp3^+ ^Tregs, which had been observed before the mogamulizumab administration, markedly diminished. Depletion of Tregs by mogamulizumab might increase activated cytotoxic T cells and cause CARs.

The use of mogamulizumab for patients who have possibility to receive allo‐HSCT should be carefully decided owing to the high incidence of GVHD. However, smoldering type ATLL patients with refractory specific skin lesions can be treated with mogamulizumab when they have no plan to receive allo‐HSCT due to age or other reasons.

## CONFLICT OF INTEREST

The authors have no conflict of interests to disclose.

## AUTHOR CONTRIBUTION

YN, KM, TK, NH, RN, and MA: made substantial contributions to conception and design. YN, KM, TK, NH, RN, and MA: treated the patient. YN, KM, and MA: drafted the manuscript. KM, TK, NH, RN, and MA: revised the manuscript critically for intellectual content.

## ETHICAL APPROVAL

This case report has been approved by the Ethics Committee of our institution. All the authors have significantly contributed to the manuscript and have approved the final version.

## INFORMED CONSENT

Written informed consent was obtained from the patient for publication of this case report.
